# One-Step Preparation of Chitosan-Based Magnetic Adsorbent and Its Application to the Adsorption of Inorganic Arsenic in Water

**DOI:** 10.3390/molecules26061785

**Published:** 2021-03-22

**Authors:** Zhe Jiang, Nian Li, Pei-Ying Li, Bo Liu, Hua-Jie Lai, Tao Jin

**Affiliations:** 1 School of Materials Science and Optoelectronic Technology, University of Chinese Academy of Sciences, Beijing 10049, China; jiangzhe18@mails.ucas.ac.cn (Z.J.); linian19@mails.ucas.ac.cn (N.L.); lipeiying19@mails.ucas.ac.cn (P.-Y.L.); laihuajie@gic.ac.cn (H.-J.L.); 2 Guangzhou Institute of Chemistry, Chinese Academy of Sciences, Guangzhou 510650, China; 3 CAS Testing Technical Services (Guangzhou) Co. Ltd., Guangzhou 510650, China; 4 Guangdong Provincial Key Laboratory of Organic Polymer Materials for Electronics, Guangzhou 510650, China; 5 CAS Engineering Laboratory for Special Fine Chemicals, Guangzhou 510650, China

**Keywords:** chitosan, adsorption, magnetic adsorbents, inorganic arsenic

## Abstract

Chitosan is a kind of biodegradable natural polysaccharide, and it is a very promising adsorber material for removing metal ions from aqueous solutions. In this study, chitosan-based magnetic adsorbent CMC@Fe_3_O_4_ was synthesized by a one-step method using carboxymethyl chitosan (CMC) and ferric salts under relatively mild conditions. The Fe_3_O_4_ microspheres were formed and the core–shell structure of CMC@Fe_3_O_4_ was synthesized in the meantime, which was well characterized via SEM/TEM, XRD, VSM, FT-IR, thermo gravimetric analysis (TGA), XPS, size distribution, and zeta potential. The effects of initial arsenic concentration, pH, temperature, contact time, and ionic strength on adsorption quantity of inorganic arsenic was studied through batch adsorption experiments. The magnetic adsorbent CMC@Fe_3_O_4_ displayed satisfactory adsorption performance for arsenic in water samples, up to 20.1 mg/g. The optimal conditions of the adsorption process were pH 3.0, 30−50 °C, and a reaction time of 15 min. The adsorption process can be well described by pseudo-second-order kinetic model, suggesting that chemisorption was main rate-controlling step. The Langmuir adsorption model provided much higher correlation coefficient than that of Freundlich adsorption model, indicating that the adsorption behavior is monolayer adsorption on the surface of the magnetic adsorbents. The above results have demonstrated that chitosan-based magnetic adsorbent CMC@Fe_3_O_4_ is suitable for the removal of inorganic arsenic in water.

## 1. Introduction

Arsenic is one of the common components of the Earth’s crust and an important pollutant in groundwater resources. At the same time, natural sources such as the dissolution of arsenic-rich rocks, volcanic emissions, fossil fuels, and garbage burning would lead to high arsenic content in water [1]. Arsenic is widely present in the water environment [2,3,4], which is also due to the widespread use of arsenic products, such as pesticides, herbicides, preservatives, etc. [5,6,7]. Exposure to elevated levels of arsenic, a class I human carcinogen, has become a global concern, affecting millions population worldwide. The currently recommended upper limit of arsenic in drinking water is 10 µg/L [8]. Arsenic exists in four oxidation states of As (V), As (III), As (0), and As (–III) [9]. The toxicity of arsenic mainly depends on its chemical form. Arsenic trivalent (As (III)) is the most toxic, followed by arsenic pentavalent (As (V)), and organic arsenic is the least toxic. Arsenic can be absorbed from groundwater by enrichment effect into soil and plants and eventually into the human body [10,11]. What is worse, long-term exposure to arsenic can result in chronic poisoning, skin lesions, neurological and respiratory defects, and even several types of cancer [12].

In consideration of the adverse impacts of metal ions on the environment, a series of treatment methods, such as chemical precipitation, ion-exchange, adsorption, membrane filtration, coagulation–flocculation, flotation, and electrochemical methods, have been proposed to remove metal ions from water samples [13]. Taking all the factors into consideration, adsorption is the better choice because of its convenience, low cost, and high efficiency [14]. Part of the heavy metal adsorbents, such as activated carbon [15], polymer ligands [16], metal oxides [17], and other nanomaterials [18], have been applied to water samples. However, some adsorbents still have low adsorption quantity, complicated production process and long separation time. Therefore, the development of adsorbents with high adsorption capacity is the key to capture metal ions effectively.

Chitosan is a deacetylated product of chitin which is widespread in the environment and it is a biodegradable natural polysaccharide. It is recognized that chitosan has many -OH and -NH_2_ groups, which have chelating effect on heavy metals under certain conditions [19,20]. In addition, -OH and -NH_2_ groups have strong reactivity, and specific groups can be introduced to enhance the adsorption of metal ions. The chelating property of chitosan has been used to adsorb metal ions, such as Mn^2+^, Hg^2+^, Pd^2+^, Cu^2+^, and Pb^2+^ [21,22].

In the present work, carboxymethyl chitosan (CMC) was modified to form a kind of magnetic adsorbent CMC@Fe_3_O_4_ via a one-step method under relatively mild conditions. In 2018, Chen et al. used a two-step synthesis method to prepare a kind of magnetic adsorbent functionalized with EDTA, which could capture anionic dye and heavy metals in complex wastewater [23]. In 2020, Lian et al. prepared a magnetic chitosan oligosaccharide and carboxymethyl cellulose nanocomposite adsorbent for Pb (II) adsorption by two-step method [24]. In comparison to other synthetic methods, the present work provided the possibility of preparing magnetic adsorbents by one-step method under relatively mild conditions. Without the steps of preparation, purification, preservation, and dispersion of Fe_3_O_4_ nanoparticles, the magnetic adsorbents could be synthesized in a relatively short period of time. The magnetic adsorbent CMC@Fe_3_O_4_ was characterized via SEM/TEM, XRD, VSM, FT-IR, TGA, XPS, size distribution, and zeta potential. Finally, the effects of adsorbent concentration, pH, temperature, contact time, and ionic strength were tested and the adsorption properties of the magnetic adsorbent CMC@Fe_3_O_4_ were evaluated by atomic fluorescence spectrometer.

## 2. Experimental Section

### 2.1. Materials and Reagents

Carboxymethyl chitosan (CMC) was bought from Shanghai Yuanye Biological Technology Co., Ltd. (Shanghai, China), and it has a degree of substitution of ≥80%. Ferric chloride (FeCl_3_·6H_2_O, 99%) and ammonia solution (25%, *v*/*v*) were purchased from Tianjin Damao Chemical Reagent Factory. Anhydrous sodium sulfite (Na_2_SO_3_, 98.0%) and glutaraldehyde (50% in H_2_O) were bought from Aladdin. A standard solution of arsenic (1000 g/L in 1 mol/L HNO_3_) was bought from National Nonferrous Metal and Electronic Materials Analysis and Testing Center. All reagents were used as received.

### 2.2. Synthesis of the Magnetic Adsorbent CMC@Fe_3_O_4_

**Scheme 1:**
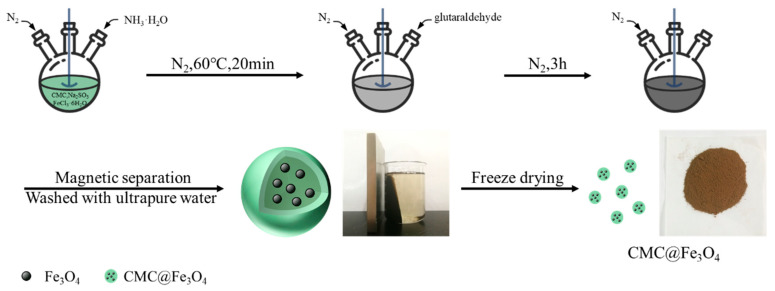
Synthesis scheme of the magnetic adsorbent CMC@Fe_3_O_4_.

The preparation of the magnetic adsorbent CMC@Fe_3_O_4_ was carried out according to previous literature with some improvements [25]. As shown in Scheme 1, 2.0 g CMC was dissolved in 200 mL ultrapure water under nitrogen gas with vigorous stirring at 60 °C. Five grams FeCl_3_·6H_2_O was dissolved in 30 mL ultrapure water and 0.72 g Na_2_SO_3_ was dissolved in 10 mL water. Then, these two solutions were mixed and poured into a separatory funnel. The mixed solution was added to the three-necked flask through funnel and stirred for 5 min. Then, 100 mL 12% NH_3_·H_2_O was dropwise added to the mixed solution through funnel. The color of the bulk solution changed from orange to black immediately. After stirring for 20 min, 20 mL 25% glutaraldehyde was added into the mixed solution without heating. The reaction pH for CMC@Fe_3_O_4_ synthesis was about 12. The reaction was finished after stirring for another 3 h at 450 r/min. The magnetic precipitates were separated by centrifugation and the supernatant was removed by magnetic separation. Finally, the magnetic precipitates were washed several times with ultrapure water and the magnetic adsorbent CMC@Fe_3_O_4_ was obtained after freeze drying treatment.

### 2.3. Adsorption Experiments

**Scheme 2:**
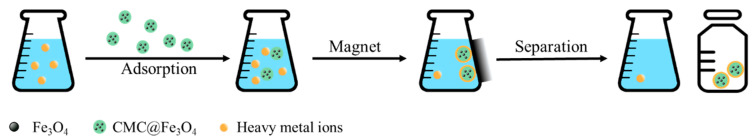
Adsorption experiments.

The effects of initial adsorbent concentration, pH, temperature, contact time, and ionic strength on the adsorption quantity were studied through batch adsorption experiments (Scheme 2). In a typical test, 50 mg magnetic adsorbent CMC@Fe_3_O_4_ was added into an arsenic solution with a concentration ranging from 2 mg/L to 90 mg/L. After oscillating in water bath for a period of time, the adsorbents were separated by a piece of magnet and the reducing agent was added to the supernatant to a constant volume of 5 mL and then taken to test the remaining arsenic concentration by atomic fluorescence spectrophotometer. The concentration results were calculated to obtain the adsorption quantity of arsenic.

### 2.4. Characterization of Magnetic Adsorbents

The magnetic adsorbent CMC@Fe_3_O_4_ was characterized via scanning electron microscope (SEM)/transmission electron microscope (TEM), X-ray diffraction (XRD), Vibrating sample magnetometer (VSM), Fourier transform infrared spectroscopy (FT-IR), Thermo gravimetric analysis (TGA), X-ray photoelectron spectroscopy (XPS), size distribution, and zeta potential. The FT-IR spectra of magnetic adsorbents were recorded by using an IRAffinity-1 spectrometer (Shimadzu, Kyoto, Japan). The typical XRD patterns were acquired on a Bruker D8 Advance X-ray diffractometer (Bruker Inc., Karlsruhe, Germany). The SEM images were obtained using Zeiss Sigma 500 (Carl Zeiss Co., Oberkochen, Germany). The TEM images were recorded by using a FEI Tecnai G20 microscope (FEI Co., Hillsboro, OH, USA). The XPS spectra of the adsorbents were performed by using a Thermo Fisher Scientific K-Alpha X-ray photoelectron spectroscopy apparatus. The magnetization curves were measured by utilizing a MPMS XL-5 vibrating sample magnetometer (Quantum Design, Inc., San Diego, CA, USA). TGA analysis was conducted via a Mettler Thermo Gravimetric instrument (Mettler Toledo Group., Columbus, OH, USA) under a N_2_ atmosphere. Malvern 3600 Zetasizer (Malvern Instruments Ltd., Melvin city, UK) was applied to determine size distribution and zeta potential of the magnetic adsorbents.

### 2.5. Chemical Analysis

An AFS series atomic fluorescence spectrometer (Beijing Haiguang Instrument Ltd., Beijing, China) was used to measure the concentrations of arsenic in water samples. The arsenic high-intensity hollow cathode lamp was employed as the radiation sources. The instrument working conditions are listed in Table 1.

## 3. Results and Discussion

### 3.1. Characterization of Magnetic Adsorbents

**Figure 1:**
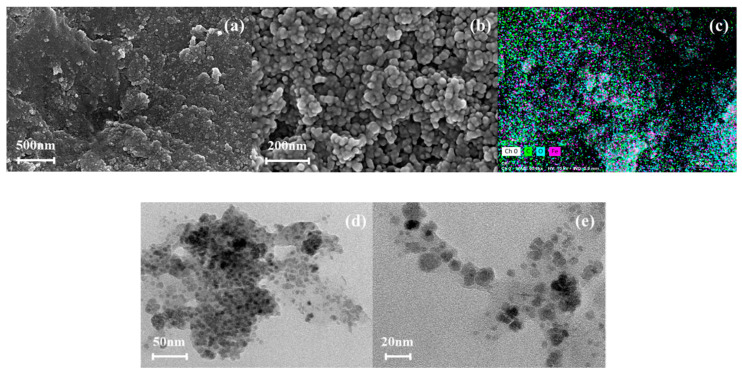
The SEM (**a**,**b**), energy-dispersive spectroscopy (EDS) (**c**), and TEM (**d**,**e**) images of the magnetic adsorbent CMC@Fe_3_O_4_.

The microstructure of the magnetic adsorbent CMC@Fe_3_O_4_ is shown in Figure 1a–c. The size of Fe_3_O_4_ magnetic nanoparticles is between 20 nm and 40 nm. Different spherical shape and irregular shape can be clearly seen under SEM, which could be speculated that organic phase has been coated on the Fe_3_O_4_ magnetic nanoparticles [22]. Because of magnetism, most of the adsorbent nanoparticles aggregate together. Simultaneously, the energy-dispersive spectroscopy (EDS) spectra were recorded to testify the composition of the magnetic adsorbents. Figure 1c shows that elements including C (15.0%), O (18.7%), and Fe (66.3%) are evenly distributed on the magnetic adsorbent CMC@Fe_3_O_4_.

The TEM images of the magnetic adsorbent CMC@Fe_3_O_4_ are shown in Figure 1d,e. The diameter of CMC@Fe_3_O_4_ is around 20 nm. The spherical shape can be clearly seen, which is consistent with the SEM results. There are dark particles coated in a large area of light gray material [23]. Combined with the SEM results, it can be concluded that the Fe_3_O_4_ nanoparticles are successfully encapsulated in carboxymethyl chitosan, which is consistent with the previous work [25].

**Figure 2:**
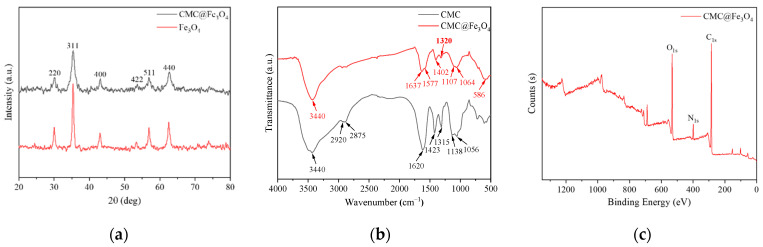
The XRD (**a**), FT-IR (**b**), and XPS (**c**) spectra images of the magnetic adsorbent CMC@Fe_3_O_4_.

In the XRD pattern of Fe_3_O_4_ (Figure 2a), six characteristic diffraction peaks appear at 30.2°, 35.6°, 43.2°, 53.6°, 57.1°, and 62.7°, which were ascribed to (220), (311), (400), (422), (511), and (440) planes of Fe_3_O_4_, respectively [26]. These peaks match well with the standard XRD pattern of Fe_3_O_4_ according to JCPDS [27]. The characteristic peaks of CMC@Fe_3_O_4_ samples appear at 30.1°, 35.4°, 43.0°, 53.5°, 56.8°, and 62.8°, which indicates that the adsorbents contain Fe_3_O_4_ nanoparticles. The results confirm that the cross-linking reaction has taken place. The FT-IR spectra of CMC and CMC@Fe_3_O_4_ is shown in Figure 2b. For CMC, the absorption bands were found at 3440 cm^−1^ (stretching vibration of N-H and O-H), 2920 cm^−1^ and 2875 cm^−1^ (stretching mode of C-H), 1620 cm^−1^ (amide), 1315 cm^−1^, and 1423 cm^−1^ (CH_3_ symmetrical angular deformation), respectively. The Fe-O bond of Fe_3_O_4_ appeared at 585 cm^−1^ in the magnetic adsorbent CMC@Fe_3_O_4_ but weakened. Thus, the CMC has been crosslinked to the surface of the Fe_3_O_4_ magnetic nanoparticles through glutaraldehyde [28]. The XPS survey spectra (Figure 2c) showed that there is no iron exposed to the surface of CMC@Fe_3_O_4_, thus the surface of the magnetic adsorbent CMC@Fe_3_O_4_ was wrapped by organic phase [29].

**Figure 3:**
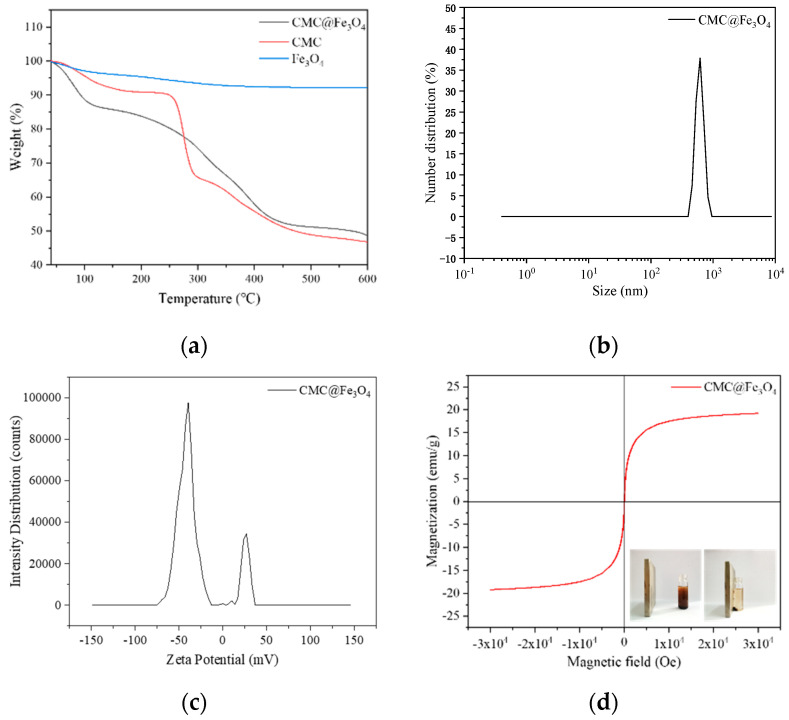
The thermo gravimetric analysis (TGA) (**a**), size distribution (**b**), zeta potential (**c**), and magnetization curves (**d**) of the magnetic adsorbent CMC@Fe_3_O_4_.

The TGA curves of the Fe_3_O_4_, CMC, and CMC@Fe_3_O_4_ samples are shown in Figure 3a. From 40 °C to 600 °C, the weight loss of Fe_3_O_4_, CMC, and CMC@Fe_3_O_4_ was 7.7%, 67.9%, and 66.4%, respectively. For Fe_3_O_4_, the total weight loss over the tested temperature is structure water and surface water. For CMC, the water loss temperature is ~245 °C, and when the temperature reaches at 295 °C, most of the organic matter begins to decompose thermally. For the magnetic adsorbent CMC@Fe_3_O_4_, a weight loss of 66.4% over the tested temperature indicates the lost weight of cross-linked CMC in the magnetic adsorbent [23]. In comparison with CMC, the magnetic adsorbent CMC@Fe_3_O_4_ showed less thermogravimetric loss, which can also prove that CMC is successfully cross-linked to Fe_3_O_4_ nanoparticles.

The size distribution and zeta potential curves of the magnetic adsorbent CMC@Fe_3_O_4_ are shown in Figure 3b,c. The particle diameter of CMC@Fe_3_O_4_ shows a concentrating distribution between 459 nm to 825 nm, which is somewhat different from the results of SEM/TEM. It is speculated that the presence of magnetism leads to the aggregation of the adsorbents which results in the increase of particle size. The zeta potential curve showed that most of the adsorbent particles had negative electronegativity, which was conducive to the adsorption of metal cations.

Magnetic hysteresis loop of the magnetic adsorbent CMC@Fe_3_O_4_ is shown in Figure 3d. The saturation magnetization value was 19.2 emu/g for CMC@Fe_3_O_4_, suggesting that the adsorbent was superparamagnetic [30], and it was easy to isolate from solution by gravity and magnetism for only a few seconds.

### 3.2. Adsorption of Arsenic on CMC@Fe_3_O_4_

**Figure 4:**
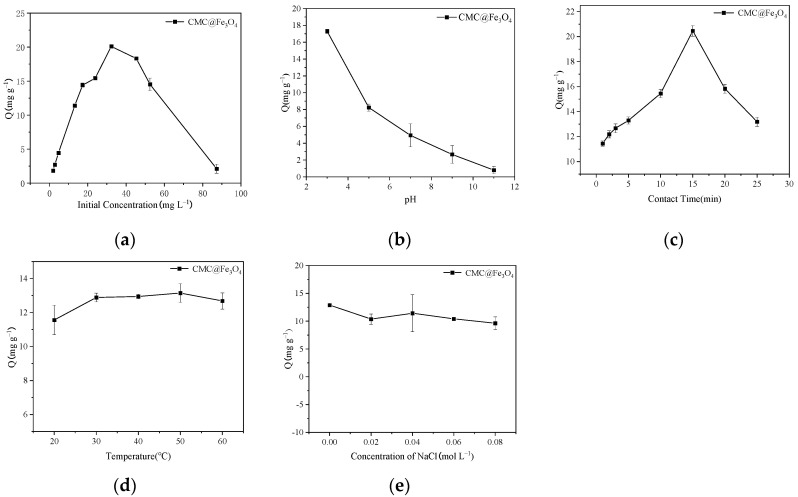
Effect of initial arsenic concentration (**a**), pH (**b**), reaction time (**c**), temperature (**d**), and ionic strength (**e**) on the adsorption quantity (Q) of arsenic by CMC@Fe_3_O_4_.

The effect of the initial arsenic concentration was tested with its loading ranging from 2 mg/L to 90 mg/L (Figure 4a). With the increase of initial arsenic concentration, the adsorption quantity (Q, mg/g) of arsenic showed a trend of first up and then down. As it reaches to 30 mg/L, the adsorption quantity gets its maximum at 20.1 mg/g. Therefore, the concentration of 30 mg/L arsenic was applied to the following experiments.

The effect of pH was explored, as shown in Figure 4b. With the increase of pH, the adsorption quantity of arsenic significantly decreased, suggesting that adsorption process would get better effect at lower pH. For example, the adsorption quantity reached 17.3 mg/g at pH 3.0. On account of the presence of Fe_3_O_4_ in the adsorbents, lower pH was not selected because it might cause dissolution of Fe_3_O_4_.

Figure 4c shows that the adsorption quantity of arsenic gradually increased as reaction continued, and reached its maximum value after reaction for 15 min. The optimal adsorption quantity was 20.8 mg/g, and it decreased significantly as the reaction time was prolonged. Therefore, 15 min is the optimal contact time. Figure 4d shows that too high or too low temperature is not conducive to the adsorption process. When the temperature was 50 °C, the adsorption quantity of arsenic reached the optimal value of 13.2 mg/g. Figure 4e showed that the ionic strength (NaCl) affects the adsorption of arsenic. The adsorption quantity declined slightly with the addition of NaCl raising from 0 to 0.08 mol/L, indicating that the presence of NaCl inhibited the absorption of arsenic.

Compared with other magnetic adsorbents or chitosan-based adsorbents, the prepared CMC@Fe_3_O_4_ adsorbents have higher adsorption quantity as shown in Table 2. As the magnetic adsorbent was prepared by a one-step process, much preparation time can be saved.

### 3.3. Effect of Contact Time on Adsorption and Kinetics Study

**Figure 5:**
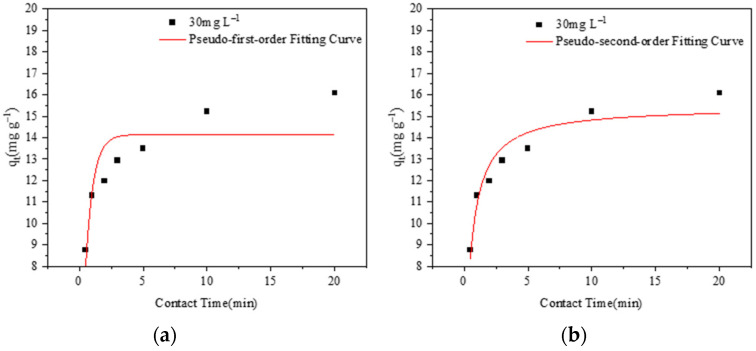
Effect of contact time on adsorption and pseudo-first-order kinetics model (**a**) and pseudo-second-order kinetics model (**b**) of arsenic at 30 mg L^−1^. Experimental conditions: [CMC@Fe_3_O_4_] = 50 mg; T = 30 °C; pH = 3.0; V = 50 mL.

The adsorption kinetics of CMC@Fe_3_O_4_ is shown in Table 3 and Figure 5. As can be seen from the figure, the adsorption gradually approached equilibrium with the increase of time, and the adsorption equilibrium could be reached after 15 min. The pseudo-first-order kinetics model and pseudo-second-order kinetics model were used for data fitting analysis, and relevant parameters were calculated by using Equations (1) and (2), respectively.

(1) $$q_{t}=q_{e}\left(1-e^{-k_{1}t}\right)\;$$

(2) $$\frac{t}{q_{t}}=\frac{1}{q_{e}}t+\frac{1}{k_{2}q_{e}^{2}}$$

where *k*_1_ is the pseudo-first-order adsorption constant (min^−1^), and *k*_2_ is the pseudo-second-order adsorption constant (g mg^−1^ min^−1^), respectively. Moreover, *q_t_* and *q_e_* represent the unit adsorption capacity at time *t* and at equilibrium adsorption (mg g^−1^), respectively.

In comparison to the pseudo-first-order kinetics model, the correlation coefficient R_2_ of the pseudo-second-order kinetic model is 0.919, and the theoretical maximum adsorption capacity (*q_e_*^cal^) calculated by the pseudo-second-order kinetic model is close to the experimental results (*q_e_*^exp^). Therefore, the adsorption process can be well described by pseudo-second-order kinetic model, suggesting that chemisorption was main rate-controlling step [35].

### 3.4. Adsorption Isotherms and Adsorption Mechanism

**Figure 6:**
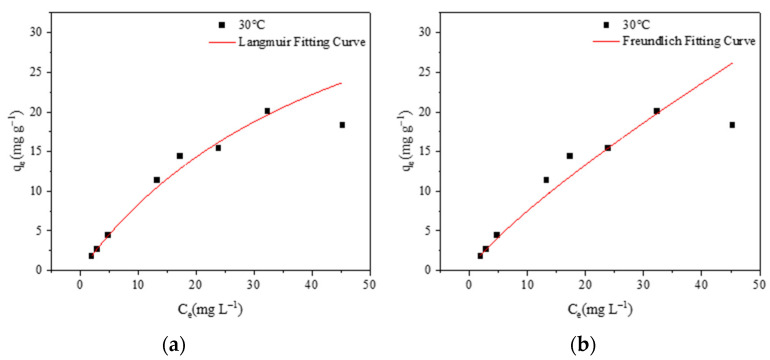
Langmuir (**a**) and Freundlich (**b**) parameters for adsorption of arsenic on CMC@Fe_3_O_4_. Experimental conditions: [CMC@Fe_3_O_4_] = 50 mg; T = 30 °C; pH = 3.0; V = 50 mL.

Figure 6 is the adsorption isotherm of CMC@Fe_3_O_4_ for arsenic at 30 °C. The adsorption amount increased with the increasing of the initial concentration, and gradually reach saturation adsorption. The saturated adsorption capacity of CMC@Fe_3_O_4_ was 20.1 mg g^−1^ at 30 °C. Langmuir and Freundlich adsorption models were used to simulate the adsorption isotherm, and the relevant parameters were calculated by Equations (3) and (4), respectively (Table 4).

(3) $$q_{e}=\frac{q_{max}K_{L}C_{e}}{1+K_{L}C_{e}}$$

(4) qe=KLCe1n

In the formula, *C_e_* (mg L^−1^) represents equilibrium concentration in aqueous solution, *q_e_* (mg g^−1^) is adsorption amount of pollutant at equilibrium, and *q_max_* (mg g^−1^) is the maximum uptake capacity of the adsorbent. *K_L_* (L mg^−1^) is a Langmuir constant concerning adsorption energy and *K_F_* represents Freundlich constant associated with sorption intensity and sorption capacity.

The related parameters show that the Langmuir adsorption model can better fit the adsorption of arsenic for CMC@Fe_3_O_4_. The Langmuir adsorption model provided much higher correlation coefficients (R^2^), indicating that the adsorption behavior is monolayer adsorption on the surface of the magnetic adsorbents. The underlying adsorption mechanism might be that the surface of carboxymethyl chitosan contains a large number of carboxyl groups, which have electrostatic attraction with the heavy metal ions with positive charge. Therefore, the CMC@Fe_3_O_4_ magnetic adsorbents could achieve the purpose of removing inorganic arsenic in water [34,36]. The best pH of the prepared CMC@Fe_3_O_4_ magnetic adsorbent is about 3. Therefore, it will inevitably lead to the dissolution of a part of the magnetic adsorbents in the process of adsorption. Recycling and reusing for three times, the adsorption quantity reduced by 20–30%.

## 4. Conclusions

In this study, chitosan-based magnetic adsorbent CMC@Fe_3_O_4_ was prepared by glutaraldehyde cross-linking reaction coupled with one-step synthesis. The as-prepared magnetic adsorbent CMC@Fe_3_O_4_ had the core–shell structure with saturation magnetization value of 19.2 emu/g. The batch adsorption experiments showed that the maximum adsorption quantity of arsenic in water was 20.1 mg/g by using 30 mg/L CMC@Fe_3_O_4_. The optimal conditions of the adsorption process were pH 3.0, 30−50 °C and a reaction time of 15 min. The ionic strength inhibited the adsorption process. The adsorption process can be well described by pseudo-second-order kinetic model, suggesting that chemisorption was main rate-controlling step. The Langmuir adsorption model provided much higher correlation coefficient than that of the Freundlich adsorption model, indicating that the adsorption behavior is monolayer adsorption on the surface of the magnetic adsorbents. Additionally, the developed CMC@Fe_3_O_4_ has the advantages of simple operation and rapid adsorption and showed satisfactory removal performance of arsenic in water samples. This study will offer good prospects to the synthesis of the adsorption materials for treating real wastewater with metal ions.

## Figures and Tables

**Table 1:** Parameters and operating conditions of atomic fluorescence spectrometer.

Parameters	Settings
Lamp current	60 mA
High negative voltage of photomultiplier	260 V
Carrier argon flow rate	300 mL/min
Shield gas flow rate	800 mL/min
HCl carrier solution	5%, *v*/*v*

**Table 2:** Comparison of adsorption capacity of CMC@Fe_3_O_4_ with different magnetic or chitosan-based adsorbents.

Magnetic Adsorbents	Q (mg g^−1^)	Reference
Iron-impregnated chitosan granular	22.5	[31]
TiO2-impregnated chitosan bead	2.1	[32]
Iron oxide-coated sponge	4.5	[33]
Iron oxide-coated cement	0.7	[34]
CMC@Fe_3_O_4_	20.1	Our work

**Table 3:** Parameters of CMC@Fe_3_O_4_ removing arsenic at 30 mg L^−1^ based on pseudo-first-order and pseudo-second-order kinetics model.

C_0_/ (mg L^−1^)	*q_e_*/ (mg g^−1^)	Pseudo-First-Order Model			Pseudo-Second-Order Model		
		*k*_1_/min^−1^	*q_e_*^cal^/(mg g^−1^)	R^2^	*k*_2_/(g mg^−1^ min^−1^)	*q_e_*^cal^/(mg g^−1^)	R^2^
30	20.1	1.7	14.1	0.727	0.15	15.5	0.919

**Table 4:** Langmuir and Freundlich parameters for adsorption of arsenic on CMC@Fe_3_O_4_.

Sample	Temperature/°C	Langmuir Model			Freundlich Model		
		*q_max_*/(mg g^−1^)	*K_L_*/(L mg g^−1^)	R^2^	*K_F_*/(g mg^−1^ min^−1^)	*n*/(mg g^−1^)	R^2^
CMC@Fe_3_O_4_	30	49.2	0.02	0.995	1.1	1.2	0.985

## Data Availability

Not applicable.

## Sample Availability

Samples of chitosan-based magnetic adsorbent CMC@Fe3O4 are available from the authors.
